# A study of factors that predict novice nurses’ perceived ability to provide care in acute situations

**DOI:** 10.1002/nop2.871

**Published:** 2021-04-02

**Authors:** Anders Sterner, Nerrolyn Ramstrand, Lina Palmér, Magnus Andersson Hagiwara

**Affiliations:** ^1^ Faculty of Caring Sciences, Work Life and Social Welfare University of Borås Borås Sweden; ^2^ Department of Rehabilitation School of Health Sciences Jönköping University Jönköping Sweden; ^3^ Faculty of Caring Science, Work Life and Social Welfare Centre for Prehospital Research University of Borås Borås Sweden

**Keywords:** acute care, clinical judgement, experience, graduate nurse, novice nurse

## Abstract

**Aims:**

To explore factors that predict novice nurses’ trust in their ability to provide care in acute situations and identify factors that are related to their perceived ability to make clinical judgements in acute situations.

**Design:**

Exploratory cross‐sectional study.

**Methods:**

Novice nurses employed within somatic care in Swedish hospitals completed an online survey. Univariate analysis facilitated exploration of the data and identification of predictor variables with the greatest association with: (1) trust in their own ability (one item) and (2) ability to make clinical judgements (four items). Multivariate binary logistic regression modelling was used to model the likelihood of outcomes based on each predictor variable.

**Results:**

The two most important predictors related to trust in ability to provide care were duration of work experience and participation in acute situations during nursing education. For clinical judgement, duration of work experience was significant in all four models and experience of acute situations post‐graduation was significant in two models.

## 1 INTRODUCTION

Internationally, as well as in Sweden, newly qualified nurses report that they are not fully prepared for working within their chosen profession (Gardiner & Sheen, 2016; Hickerson et al., 2016; Widarsson et al., 2020). One area that has been described as particularly complex for novice nurses is acute care settings (Duchscher, 2009; Hawkins et al., 2019). Acute situations are characterized by sudden events that must be attended to rapidly and without hesitation while subsequent decisions regarding courses of action must be taken without delay and can have serious consequences (Benner, 2001).

To facilitate development of interventions aimed at improving the competence of newly graduated nurses in acute situations, it is necessary to identify factors that are associated with their ability to provide care.

## 2 BACKGROUND

Acute care requires effective management of rapidly changing situations and necessitates that nurses identify problems promptly, intervene appropriately and assess and mobilize help (Benner, 2001). Sterner et al. (2018) studied novice nurses’ perception of acute situations in acute care settings, concluding that they are characterized by more than a medical manifestation related to a specific patient. In addition to a sudden occurrence, the authors suggest that acute situations, as perceived by novice nurses, are situations in which one's competence is perceived as inadequate, responsibility considered overwhelming and in which there is insufficient time in relation to the tasks that must be performed. An integrative review found that the dominating concept describing nurses’ transition to acute care was fear. Fear of higher acuity patients, making mistakes, potentially harming patients and not being able to meet expectations (Hawkins et al., 2019). One reason for this fear could be that novice nurses are expected to provide care in acute situations with the same level of competence as an experienced nurse, to interpret complex findings and to decide upon appropriate actions in a timely manner (Butler, 2018).

A systematic review investigating factors affecting the development of nurse competencies found that work experience and educational level influenced competence (Rizany et al., 2018). Undergraduate nurse education (Massey et al., 2017; Purling & King, 2012; Sterner et al., 2019) and prior experience in managing acute situations have also been suggested as important factors affecting nurses’ ability to provide adequate care (Butler, 2018; Massey et al., 2017; Sterner et al., 2019). While there are many examples of studies that have investigated factors affecting nurse competence, few have specifically targeting nurse competence in acute situations.

Nursing education has been described as insufficient in providing appropriate experience in performing care in acute situations (Herron, 2018; Smith & Rushton, 2018). Benner's novice to expert model (Benner, 1982) describes experience as more than the mere passage of time, suggesting that it is a base for acquisition and development of skill performance in clinical nursing. The model postulates that nurses pass through five levels of proficiency, ranging from reliance on abstract principles to the application of concrete experiences (Benner, 1982). Benner (2001) claims that most newly graduated nurses are novices or, at best, advanced beginners, who can barely demonstrate acceptable care. She suggests that experience in the types of situations that they are expected to perform in the workforce are a major factor in the development of expertise.

Clinical decisions or clinical judgements are often complex and are essential in situations that are ambiguous (Tanner, 2006). As such, clinical decision‐making is regarded as a fundamental skill for all nurses (Bucknall et al., 2016). Tanner (2006) has developed a clinical judgement model which aims to describe how nurses think when they are engaged in clinical situations that require judgment. The model emphasizes the nurses’ role of noticing, interpreting and responding. Noticing, gaining an initial grasp of the situation, is influenced by textbook knowledge and experience of similar patients. The act of noticing will then trigger one or more reasoning patterns, which help the nurse to interpret the meaning of data they observe and collect and to determine the appropriate response.

Beyond experience and adequate decision‐making skills, there is little knowledge of specific factors that influence novice nurses’ ability to provide care in acute situations. As such, more detailed knowledge is required to facilitate development of interventions that promote graduate nurses ability to care in acute situations.

The aims of the present study were to explore:

‐ Factors that predict novice nurses’ trust in their ability to provide care in acute situations.
‐ Factors that predict novice nurses’ perceived ability to make clinical judgements in acute situations.

## 3 METHOD

### 3.1 Study design

The study used an exploratory cross‐sectional design based on an online survey.

### 3.2 Participants

To be included in the study participants were required to have less than one year of work experience as a nurse and to be employed, as a nurse, in a hospital providing somatic care for both adults and/or children. The definition of novice nurses as having less than one year of work experience was based upon Benner's description of novice nurses (Benner, 1982, 2001).

### 3.3 Setting

**FIGURE 1:**
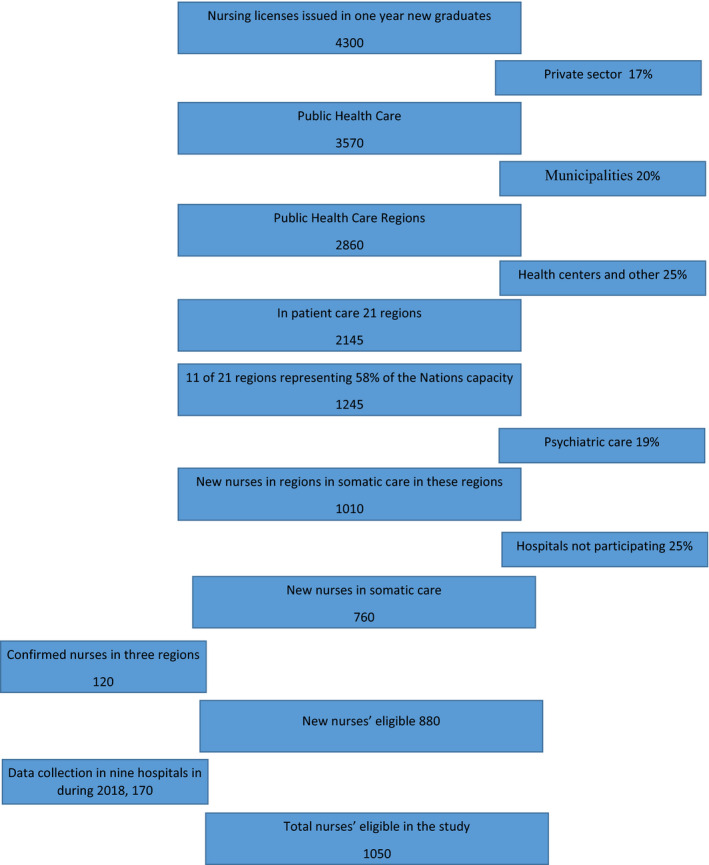
Nurses eligible to participate in the study

This study was conducted with staff from county and university hospitals in Sweden. As this was an exploratory study, there was no hypothesis regarding potential outcomes and a sample size calculation was not possible. Representatives employed within hospitals, and who had direct contact with novice nurses (e.g., Human resources personnel, nurse introductory program coordinators) were requested to assist with recruitment of novice nurses for the study. Representatives were approached from each of Sweden's 21 healthcare regions and were requested to e‐mail information, as well as a link to the online survey, to nurses who met the inclusion criteria. Hospital representatives were also requested to send a reminder 1–2 weeks after the initial e‐mail. In the e‐mail to nurses, information about the study and contact details to the research group were provided. As representatives were not all able to specifically identify novice nurses working in somatic care, a question was included in the survey to identify this group from those working in other areas. The total number of novice nurses who met the inclusion criteria and received an invitation to participate in the study is unknown. In 2018, the Swedish National board of Health and Welfare registered 4,300 newly graduated nurses in Sweden (National Board of Health & Welfare, 2019). The proportion of these who went on to enter the workforce as practicing nurses is unclear however, our estimation is that 1,050 nurses were eligible to participate in this study. The calculation for this is presented in Figure 1 as supplementary information and discussed in detail in the limitations section of this manuscript.

### 3.4 Data collection

Data were collected using an online survey and in parallel with another instrument design study (Sterner et al., 2020). Questions were in Swedish and are described below. In order to enhance face and content validity of the questions, they were initially reviewed by an expert panel and then through the use of cognitive interviews. The expert panel comprised of four registered nurses with knowledge and experience of acute care. Two of them held a doctoral degree. The panel of experts were requested to independently assess the relevance of specific questions and the structure of the scale. Cognitive interviews were performed with two novice nurses working in acute care at different hospitals and wards. During cognitive interviews participants were requested to “think aloud” when completing the survey. The interviewer also asked for information relevant to the question or answer given. Prior to completing the online survey, participants were provided with the following definition of acute situations, which is grounded in previously published work (Sterner et al., 2018): *Situations that novice nurses themselves perceived as acute. Including situations in which something happens suddenly, and the care situation changes, or the perception that there is insufficient time in relation to the tasks that must be performed*.

The online survey that was used to collect data for this study included questions related to participant demographics, as well as questions related to novice nurses’ perceived ability to provide care in acute situations. Demographic data included questions related to sex and age, as well as background data related to prior education in another health‐related professions, work experience in health care before and during nursing education, participation in acute situations during nursing education, experience of acute situations post nursing education, duration of work experience as well as the place and department in which they worked. The choice of background data to collect was based upon work by Benner (1982), Tanner (2006) and Lasater et al. (2015), which identified experience as a necessary component for developing an ability to make rapid and appropriate clinical judgements in acute situations.

Questions related to novice nurses perceived trust in their ability to provide care in acute situations and their perceived ability to make clinical judgement in acute situations served as response variables and included five questions, these have been translated into English and presented in Table 1. These questions were not part of the instrument which was developed during same data collection process and reported in a previous publication (Sterner et al., 2020). Responses were collected on a 4‐point scale ranging from strongly disagree to strongly agree or very poor to very good. An even number of response variables was chosen to force respondents to clearly express their opinion (Streiner et al., 2015).

**TABLE 1:** Response variables with questions

Response variables	Questions
Trust in ability to care in acute situations	I trust my ability to provide care in acute situations
Clinical judgement	I estimate my ability to realise when a patient is in an acute situation as:
	I estimate my ability to independently determine necessary actions in acute situations as:
	I estimate my ability to independently prioritise between actions in acute situations as:
	I estimate my ability to independently take action in acute situations as:

Data collection was anonymous and performed using Sunset Survey software (version 4.3.9.5, Artisan Global Media). Data were collected from late 2018 until early 2019.

### 3.5 Statistical analysis

For demographic data, descriptive statistics were applied and binary logistic regression modelling was used to explore the relationship between predictors and the response variables shown in Table 1 (Polit & Beck, 2009; Sperandei, 2014). Predictors used in the regression analysis included sex, education in health care before nursing education, work experience in health care before nursing education, work experience in health care during nursing education, participation in an acute situation during nursing education, work experience as a nurse (months) and experience of acute situations. Response variables are presented in Table 1.

The four‐point responses on outcome variables were dichotomized for analysis. For example, in the question “I trust my ability to provide care in acute situations”, the response options of: strongly disagree, somewhat agree, agree and strongly agree were dichotomized into disagree and agree. Response variables very poor, poor, good and very good were dichotomized into poor and good (Sterner et al., 2020).

Logistic regression transforms the probability of an event occurring into odds and odds ratios (OR). As logistic regression is sensitive to extremely high correlations between predictor variables (Tabachnick & Fidell, 2013), bivariate correlations were initially performed using Spearman Rho. A cut‐off value was established at ≥0.80. Since the correlation calculation did not indicate a serious concern regarding multi‐collinearity (Midi et al., 2010) all predictor variables were included in the regression analysis.

Univariate model testing of the predictors was performed and a *p*‐value ≤ 0.25 was considered appropriate for including them in multivariate modelling (Bursac et al., 2008; Sperandei, 2014). Binary logistic regression modelling, using enter instruction (direct), was applied to ensure that all predictor variables were entered the logistic regression equation simultaneously (Tabachnick & Fidell, 2013). Model fit was estimated by the Hosmer and Lemeshow test of goodness of fit (*p* > 0.05) (Tabachnick & Fidell, 2013). Effect size was calculated using Nagelkerke's *R* ^2^ (Harrell, 2015; Tabachnick & Fidell, 2013).

Descriptive data, frequency statistics, correlation assessment and binary logistic regressions were analysed using IBM statistics SPSS version 25 for windows (IBM Corp, 2013).

### 3.6 Ethical considerations

This study did not involve patients, relatives or sensitive personal information. According to the Swedish Ethical Review Act (SFS 2003:460, 2003), no ethical approval was required. The research was, however, conducted in accordance with the requirements stated in the Declaration of Helsinki (World Medical Association, 2013). This has been done by ensuring that participation in the study was voluntary and that participants’ responses were anonymous. Consent to participate was assumed if nurses chose to complete and submit the survey. The collected data were stored in a password protected database and data could not be linked to a specific participant.

## 4 RESULTS

### 4.1 Results from the survey process

Representatives from 14 healthcare regions in Sweden agreed to contact nurses who met the inclusion criteria within their respective regions. The sample included 33 hospitals (both county and university hospitals). A total of 227 participants completed the questionnaire. Eighteen participants were excluded as they did not fulfil the inclusion criteria of working in somatic care or having worked as a nurse for less than 1 year. A description of participants and demographics of the remaining 209 participants are presented in Table 2.

**TABLE 2:** Participant demographics

Variable	Value
Participants	209
Gender, *N* (%)	
Female	175 (83.7)
Male	33 (15.8)
Other	1 (0.5)
Median age (range)	26 (22–54)
Median months working experience (range)	6 (1–12)
Work experience in health care before nursing education, *N* (%)	132 (63)
Education in another health care related profession before nursing education, *n* (%)	76 (36)
Work experience in health care during nursing education, *N* (%)	197 (94)
Experience of acute situations during nursing education, *N* (%)	136 (65)
Experience of acute situations post education, *N* (%)	
Few	161 (77)
Many	48 (23)
Workplace	
Accident and emergency dep, *N* (%)	25 (12)
Medical, *N* (%)	75 (36)
Surgical, Orthopaedic, *N* (%)	63 (30)
Other/combination, *N* (%)	46 (22)

### 4.2 Factors that predict novice nurses trust in their ability to provide care in acute situations

The univariate analysis of predictor variables resulted in six variables that were considered appropriate for inclusion in the multivariate model. All predictor variables that were considered appropriate for modelling are presented in Table 3.

**TABLE 3:** Univariate modelling *p*‐value ≤ 0.25

	*B*	*SE*	Wald	*df*	Sig.	OR	95% CI	
I trust my ability to provide care in acute situations								
Sex (Male)	−0.828	0.386	4.599	1	0.032	0.437	0.205	0.931
Education in health care before nursing education	0.619	0.304	4.133	1	0.042	1.856	1.022	3.37
Participation in acute situation during nursing education	1.239	0.361	11.783	1	0.001	3.452	1.701	7.002
Working experience (months)	0.147	0.046	10.083	1	0.001	1.159	1.058	1.269
Experience of acute situations since nursing education	1.125	0.34	10.936	1	0.001	3.08	1.581	5.998
Workplace: Accident and emergency dep.			13.944	3	0.003			
Workplace (1) Medical	−1.186	0.478	6.153	1	0.013	0.306	0.12	0.78
Workplace (2) Surgical, Orthopaedic	−1.688	0.515	10.742	1	0.001	0.185	0.067	0.507
Workplace (3) Other/combination	−0.504	0.501	1.011	1	0.315	0.604	0.226	1.613

Abbreviation: OR, Odds Ratio.

A multivariate binary logistic regression was performed to determine the influence of predictor variables on each response variable. The Hosmer and Lemeshow test indicated a good model fit *p* = 0.845. The model explained 27% (Nagelkerke *R* ^2^) of the variance related to trust in ability to provide care in acute situations. The model classified 73% of cases and indicated that duration of work experience (OR 1.15, 95% 1.0–1.3, *p* < 0.01), participation in acute situations during nursing education (OR 3.05, 95% 1.4–6.6, *p* < 0.005), education in a health profession before nursing education (OR 1.75, 95% 1.1–4.6, *p* < 0.025) and working in accident and emergency department were positively and independently associated with the perception of trust in ability to care in acute situations. Females (OR 0.40, 95% 0.16–0.99, *p* < 0.04) were negatively and independently associated with the perception of trust in ability to provide care in acute situations. Full models are presented in Table 4.

**TABLE 4:** Full model: I trust my ability to provide care in acute situations

	*B*	*SE*	Wald	*df*	Sig.	OR	95% CI	
I trust my ability to provide care in acute situations								
Sex (Male)	−0.904	0.457	3.909	1	**0.048**	0.405	0.165	0.992
Education in health care before nursing education	0.818	0.364	5.044	1	**0.025**	2.265	1.11	4.623
Participation in acute situation during nursing education	1.115	0.395	7.95	1	**0.005**	3.049	1.405	6.617
Working experience (months)	0.14	0.054	6.663	1	**0.01**	1.15	1.034	1.278
Experience of acute situations since nursing education	0.562	0.412	1.861	1	0.173	1.754	0.782	3.933
Workplace: Accident and emergency dep.			9.237	3	**0.026**			
Workplace (1) Medical	−0.738	0.548	1.809	1	0.179	0.478	0.163	1.401
Workplace (2) Surgical, Orthopaedic	−1.119	0.6	3.479	1	0.062	0.326	0.101	1.059
Workplace (3) Other/combination	0.227	0.589	0.149	1	0.7	1.255	0.396	3.98
Constant	−0.725	1.003	0.522	1	0.47	0.484		

Note

Bold *p* ≤ 0.05

### 4.3 Factors that predict novice nurses’ perceived ability to make clinical judgements in acute situations

Univariate testing against the four response variables related to the second aim of the study resulted in eight predictor variables considered appropriate for modelling. All predictor variables were considered appropriate and are presented in Table 5.

**TABLE 5:** Univariate modelling *p*‐value ≤ 0.25

	*B*	*SE*	Wald	*df*	Sig.	OR	95% CI	
Ability to realize when a patient is in an acute situation								
Working experience in health care before nursing education	0.573	0.383	2.246	1	0.134	1.774	0.838	3.756
Work experience in health care during nursing education	1.84	0.613	8.998	1	0.003	6.296	1.892	20.95
Participation in acute situation during nursing education	0.675	0.384	3.098	1	0.078	1.965	0.926	4.169
Working experience (months)	0.215	0.066	10.76	1	0.001	1.24	1.09	1.41
Experience of acute situations since nursing education	1.702	0.749	5.157	1	0.023	5.485	1.262	23.83
Workplace: Accident and emergency dep.			1.584	3	0.663			
Workplace (1) Medical	−0.784	0.802	0.957	1	0.328	0.457	0.095	2.197
Workplace (2) Surgical, Orthopaedic	−0.995	0.804	1.533	1	0.216	0.37	0.076	1.787
Workplace (3) Other/combination	−0.725	0.844	0.738	1	0.39	0.484	0.093	2.532
Ability to independently determine necessary actions								
Working experience in health care before nursing education	0.474	0.297	2.559	1	0.11	1.607	0.899	2.874
Education in health care before nursing education	0.486	0.309	2.479	1	0.115	1.626	0.888	2.978
Work experience in health care during nursing education	0.977	0.604	2.612	1	0.106	2.656	0.812	8.683
Participation in acute situation during nursing education	0.434	0.299	2.1	1	0.147	1.543	0.858	2.775
Working experience (months)	0.286	0.052	29.69	1	0	1.331	1.201	1.475
Experience of acute situations since nursing education	1.455	0.439	10.979	1	0.001	4.283	1.812	10.13
Workplace: Accident and emergency dep.			3.734	3	0.292			
Workplace (1) Medical	−0.519	0.527	0.968	1	0.325	0.595	0.212	1.673
Workplace (2) Surgical, Orthopaedic	−0.93	0.533	3.047	1	0.081	0.395	0.139	1.121
Workplace (3) Other/combination	−0.427	0.564	0.572	1	0.449	0.653	0.216	1.972
Ability to independently prioritize between actions								
Sex (male)	−0.61	0.455	1.799	**1**	0.18	0.543	0.223	1.325
Working experience in health care before nursing education	0.664	0.306	4.709	1	0.03	1.942	1.066	3.538
Work experience in health care during nursing education	1.211	0.606	3.99	1	0.046	3.355	1.023	11
Participation in acute situation during nursing education	0.7	0.308	5.151	1	0.023	2.013	1.1	3.684
Working experience (months)	0.189	0.049	14.753	1	0	1.208	1.097	1.331
Experience of acute situations since nursing education	1.631	0.5	10.648	1	0.001	5.109	1.918	13.61
Workplace: Accident and emergency dep.			7.64	3	0.054			
Workplace (1) Medical	−0.981	0.669	2.152	1	0.142	0.375	0.101	1.39
Workplace (2) Surgical, Orthopaedic	−1.64	0.667	6.051	1	0.014	0.194	0.053	0.717
Workplace (3) Other/combination	−1.364	0.689	3.919	1	0.048	0.256	0.066	0.987
Ability to independently take action								
Sex (male)	−0.564	0.436	1.676	1	0.195	0.569	0.242	1.336
Work experience in health care during nursing education	0.708	0.597	1.409	1	0.235	2.031	0.63	6.543
Participation in acute situation during nursing education	0.572	0.302	3.574	1	0.059	1.771	0.979	3.205
Working experience (months)	0.228	0.05	20.901	1	0	1.256	1.139	1.385
Experience of acute situations since nursing education	1.556	0.465	11.194	1	0.001	4.74	1.905	11.79
Workplace: Accident and emergency dep.			7.84	3	0.049			
Workplace (1) Medical	−1.359	0.662	4.218	1	0.04	0.257	0.07	0.94
Workplace (2) Surgical, Orthopaedic	−1.769	0.666	7.065	1	0.008	0.17	0.046	0.628
Workplace (3) Other/combination	−1.166	0.694	2.823	1	0.093	0.312	0.08	1.214

Abbreviation: OR, Odds Ratio.

Four different multivariate binary logistic regressions were performed to determine the influence of predictor variables on the four response variables. The first model investigated how predictor variables influenced novice nurses’ ability to realize when a patient is in an acute situation. The Hosmer and Lemeshow test indicated a good model fit *p* = 0.510. The model explained 22% (Nagelkerke *R* ^2^) of the variance and classified 86% of cases. The model showed that work experience during nursing education (OR 7.39, 95% 1.73–31.4, *p* < 0.007) and duration of work experience (OR 1.20, 95% 1.04–1.37, *p* < 0.008) were statistically, positively and independently associated with participants perception of their ability to realize when a patient is in an acute situation.

The second model was performed to evaluate novice nurses’ ability to independently determine necessary actions. The Hosmer and Lemeshow test indicated a good model fit *p* = 0.213. The model explained 28% (Nagelkerke *R* ^2^) of the variance and classified 76% of cases. It indicated that a longer duration of work experience (OR 1.29, 95% 1.02–8.01, *p* < 0.000) and experience of acute situations post nursing education (OR 2.86, 95% 1.02–8.01, *p* < 0.044) were statistically, positively and independently associated with nurse's perceptions of their ability to independently determine necessary actions in an acute situation.

The third model was used to evaluate novice nurse's ability to independently prioritize between actions. The Hosmer and Lemeshow test indicated a good model fit *p* = 0.975. The model explained 26% (Nagelkerke *R* ^2^) of the variance and classified 76% of cases. It showed that duration of work experience (OR 1.17, 95% 1.05–1.30, *p* < 0.003), work experience in health care before nursing education (OR 2.25, 95% 1.10–4.59, *p* < 0.025) and experience of acute situations post nursing education (OR 3.66, 95% 1.16–11.52, *p* < 0.026) were statistically, positively and independently associated with novice nurse's perception of their ability to prioritize between actions in an acute situation.

The fourth model was used to evaluate novice nurse's ability to independently act. The Hosmer and Lemeshow test indicated a good model fit *p* = 0.473. The model explained 24% (Nagelkerke *R* ^2^) of the variance and classified 74% of cases. This model showed that duration of work experience (OR 1.22, 95% 1.10–1.36, *p* < 0.000) was statistically, positively and independently associated with nurse's perception of their ability to take action in an acute situation. Full models are presented in Table 6.

**TABLE 6:** Full models

	*B*	*SE*	Wald	*df*	Sig.	OR	95% CI	
Ability to realise when a patient is in an acute situation								
Working experience in health care before nursing education	0.46	0.431	1.14	1	0.286	1.584	0.681	3.685
Work experience in health care during nursing education	2.001	0.739	7.34	1	**0.007**	7.396	1.739	31.45
Participation in acute situation during nursing education	0.523	0.423	1.531	1	0.216	1.687	0.737	3.862
Working experience (months)	0.182	0.069	6.947	1	**0.008**	1.2	1.048	1.373
Experience of acute situations since nursing education	1.798	0.927	3.762	1	0.052	6.039	0.981	37.17
Workplace: Accident and emergency dep.			0.358	3	0.949			
Workplace (1) Medical	−0.164	0.907	0.033	1	0.857	0.849	0.143	5.024
Workplace (2) Surgical, Orthopaedic	−0.341	0.92	0.138	1	0.711	0.711	0.117	4.314
Workplace (3) Other/combination	−0.023	0.955	0.001	1	0.981	0.978	0.15	6.356
Constant	−1.735	1.176	2.176	1	0.14	0.176		
Ability to independently determine necessary actions								
Working experience in health care before nursing education	0.339	0.392	0.749	1	0.387	1.404	0.651	3.029
Education in health care before nursing education	0.385	0.395	0.951	1	0.329	1.47	0.678	3.185
Work experience in health care during nursing education	0.966	0.715	1.827	1	0.176	2.628	0.648	10.67
Participation in acute situation during nursing education	0.349	0.349	1	1	0.317	1.418	0.715	2.812
Working experience (months)	0.257	0.055	21.98	1	**0**	1.294	1.162	1.441
Experience of acute situations since nursing education	1.054	0.524	4.038	1	**0.044**	2.869	1.026	8.019
Workplace: Accident and emergency dep.			1.083	3	0.781			
Workplace (1) Medical	−0.171	0.634	0.072	1	0.788	0.843	0.243	2.924
Workplace (2) Surgical, Orthopaedic	−0.449	0.646	0.483	1	0.487	0.638	0.18	2.265
Workplace (3) Other/combination	−0.013	0.675	0	1	0.985	0.987	0.263	3.705
Constant	−2.324	0.96	5.863	1	0.015	0.098		
Ability to independently prioritise between actions								
Sex (Male)	−0.601	0.516	1.36	1	0.244	0.548	0.199	1.506
Working experience in health care before nursing education	0.813	0.364	4.992	1	**0.025**	2.254	1.105	4.598
Work experience in health care during nursing education	1.135	0.703	2.609	1	0.106	3.111	0.785	12.33
Participation in acute situation during nursing education	0.575	0.346	2.765	1	0.096	1.778	0.902	3.503
Work experience (months)	0.159	0.054	8.748	1	**0.003**	1.173	1.055	1.304
Experience of acute situations since nursing education	1.298	0.585	4.932	1	**0.026**	3.662	1.165	11.52
Workplace: Accident and emergency dep.			3.537	3	0.316			
Workplace (1) Medical	−0.51	0.75	0.464	1	0.496	0.6	0.138	2.608
Workplace (2) Surgical, Orthopaedic	−1.124	0.759	2.194	1	0.139	0.325	0.073	1.438
Workplace (3) Other/combination	−0.834	0.773	1.164	1	0.281	0.434	0.095	1.976
Constant	−0.373	1.331	0.079	1	0.779	0.688		
Ability to independently take action								
Sex (Male)	−0.436	0.483	0.817	1	0.366	0.646	0.251	1.665
Work experience in health care during nursing education	0.731	0.665	1.208	1	0.272	2.078	0.564	7.653
Participation in acute situations during nursing education	0.46	0.339	1.843	1	0.175	1.585	0.815	3.081
Working experience (months)	0.207	0.053	15.26	1	**0**	1.229	1.108	1.364
Experience of acute situations since nursing education	0.931	0.524	3.153	1	0.076	2.536	0.908	7.083
Workplace: Accident and emergency dep.			3.026	3	0.388			
Workplace (1) Medical	−1.033	0.727	2.02	1	0.155	0.356	0.086	1.48
Workplace (2) Surgical, Orthopaedic	−1.216	0.736	2.727	1	0.099	0.296	0.07	1.255
Workplace (3) Other/combination	−0.82	0.763	1.154	1	0.283	0.441	0.099	1.966
Constant	0.114	1.28	0.008	1	0.929	1.121		

Note

Bold *p* ≤ 0.05

## 5 DISCUSSION

This study provides evidence of specific factors that are related to novice nurses’ perceived trust in their ability to provide care and make clinical judgements in acute situations. These factors can be categorized as occurring before, during and post nursing education and, as such, are relevant from both a nursing education and healthcare service provider perspective.

The most important factors that influence novice nurse's ability to notice, interpret and respond in acute situations were experience of acute situations post‐graduation, work experience during nursing education and duration of work experience as a nurse. The odds for the different outcomes showed an increased between 1.20–7.39. Participation in acute situations during nursing education was the most influential factor related to novice nurses trust in their own ability. This is alarming since studies have previously shown that this experience is minimal or sometimes absent during nursing education (Herron, 2018; Smith & Rushton, 2018; Sterner et al., 2019). Duration of work experience was significant in all five models. Correlations between the duration of work experience and a higher assessment of competence has been found in numerous studies using a nurse competence scale (Flinkman et al., 2017).

Experience of acute situations post nursing education was present in all five models and significant in two. This indicates the importance of personal experience and that the amount of experience with acute situations is an important factor in the ability to care in acute situations. Results are consistent with earlier research (Della Ratta, 2016; Purling & King, 2012; Sterner et al., 2018, 2019). Prior work experience in health care was significant in one model. This has earlier been associated with lower self‐reported competence, in Sweden (Wangensteen et al., 2012) and internationally (Kiekkas et al., 2019). Other studies have found an association with higher self‐reported competence (Gardulf et al., 2016). Sex was a significant variable in one model. However, males represented only 15.8% of the participants in this study so it is difficult to draw any conclusion from this finding.

This study continues to build upon Benners (Benner, 1982) evidence for experience being a key factor in the development of competencies. With increased exposure to acute situations during nursing education as well as experience employed as a nurse, nurses in this study reported a higher level of trust in their ability to provide care in acute situations.

An important competence for nurses is clinical judgment and this study confirms that nurses’ prior experience is a predictor of perceived ability to make appropriate clinical judgements in acute situations. Tanner (2006) states that clinical judgment by nurses is influenced to a greater extent by what they bring to the acute situation than data about the situation at hand, and that nurses’ experience may impact on what they see and notice as well as how they interpret this data before responding (Tanner, 2006).

As discussed earlier, the lack of experience of acute situations among newly graduated nurses is alarming. Nursing education today is inconsistent and a lottery regarding the type of experience and competence that can be gained in acute situations. This is largely due to variance in quality of clinical placements (Sterner et al., 2019). Results of this study should be seen as a challenge to nursing educationalists to explore creative solutions to provide students with experience in acute situations. This might mean that the clinics where nursing students are assigned for practical placements need to be broadened or changed. Another potential intervention is the use of simulation of acute situations during nursing education. In a systematic review and meta‐analysis, Haddeland et al. (2018) reports increased knowledge and skill performance in the care of deteriorating patients after using simulation as a tool in nursing education. An important issue to consider when developing simulation scenarios is contextualization as tasks and interactions become more realistic and natural when conducted under contextualized conditions (Engström et al., 2016). Together these aforementioned measures could be important steps in developing novice nurse's ability to provide care in acute situations.

### 5.1 Methodologic considerations

Direct logistic regression was used in this study as we did not have a specific hypotheses about the order or importance of the predictor variables (Tabachnick & Fidell, 2013). Choice of variables to include in regression models is important and specific variables used in this study were carefully considered by the research group, tested for collinearity and based on theory. There is, however, a possibility that important predictors were missed.

It is important to emphasize that the models in this study are built on self‐report data and are not measurements of the nurses’ true competence in acute situations. Self‐report data are, however, a frequently used measure of nurse competence in studies of nursing education (Lejonqvist et al., 2016) and practice (Flinkman et al., 2017). According to Bandura (2010), self‐efficacy, belief in one's ability, affects performance on a specific task. Therefore, it is of interest to investigate how nurses perceive their ability to perform tasks.

### 5.2 Limitations

The research group acknowledges some limitations in this study. One of which is that the response variables’ psychometric properties were not subject to testing. Four variables, regarding perceived ability to make clinical judgement, are however consistent with those reported by Tanner (2006). The predictor variables are not categorized according to specific type of work experience or type of education, nor the length of education. The reason for this is that there is a tremendous variation of workplaces and types of education. The length and amount of work experience is also a measure that is hard to define as many of the participants could have been hourly or seasonal employees for many years. To overcome this issue, we categorized prior work experience as simply no or yes.

We had no control of how many participants who fulfilled the inclusion criterion were invited to participate in this study. Therefore, we are unable to present any response rate data. The estimation of eligible participants presented in Figure 1 is based upon national data that show 17% of nurses in Sweden are employed within the private sector while the remaining 83% work within the public sector (National Board of Health & Welfare, 2019). Nurses in the public sector work in both regional 80% and municipal hospitals 20% (Swedish Association of Local Authorities & Regions, 2019). The region is responsible for hospitals (somatic and psychiatric orientation) in which 75% of nurses work while primary healthcare and other employers account for the remaining 25% of nurses. Psychiatric care accounts for approximately 20% of the beds in hospitals. The 33 hospitals included in this study do not include all hospitals in the regions; therefore, we calculated an additional dropout rate of eligible nurses at 25%, a conservative estimation.

## 6 CONCLUSION

A variety of factors contribute to novice nurses’ perception of trust in their ability to provide care in acute situations and their perceived ability to make clinical judgements in acute situations. These factors include events that occur prior to nurse education, during nursing education and post‐graduation. Prior experience of acute situations seems to be a particularly important factor related to novice nurses’ ability to provide care in acute situations. The result has implications for developing interventions, which can provide necessary experience in acute situations for nursing students and new graduates.

## CONFLICT OF INTEREST

Each of the authors has read the submission and declare that they have no conflicts of interest related to the study.

## AUTHOR CONTRIBUTIONS

AS: Conceptualization, Investigation, Formal analysis, Writing–Original Draft. NR: Conceptualization, Methodology, Writing–Review and Editing. LP: Writing–Reviewing and Editing. MAH: Conceptualization, Formal analysis, Writing–Reviewing and Editing, Supervision. All authors have agreed on the final version and meet at least one of the following criteria [recommended by the ICMJE (https://www.icmje.org/recommendations/)]: substantial contributions to conception and design, acquisition of data or analysis and interpretation of data; drafting the article or revising it critically for important intellectual content.

## ETHICAL APPROVAL

The study methods do not fall within the boundaries of the Swedish ethic review act 2003:460.

## Data Availability

The data that support the findings of this study are available from the corresponding author upon reasonable request.
